# Pickering emulsions stabilized by ultrasound-assisted phosphorylated cantaloupe seed protein isolate −chitosan: Preparation, characterization and stability

**DOI:** 10.1016/j.ultsonch.2025.107246

**Published:** 2025-01-28

**Authors:** Ruihua Zhang, Shuting Li, Mingyan Ai, Shenghuizi Chen, Chunlan Zhang, Zhiqiang Zhou, Lili Huang, Xiang Li, Jiankang Lu

**Affiliations:** a College of Food Science and Engineering, Tarim University, Alar 843300, China; b Production & Construction Group Key Laboratory of Special Agricultural Products Further Processing in Southern Xinjiang, Tarim University, Alar 843300, China; c Wuhan Academy of Agricultural Sciences, Wuhan 430000, China; d Instrumental Analysis Center, Tarim University, Alar 843300, China; e College of Chemistry and Chemical Engineering, Tarim University, Alar 843300, China; f Xinjiang Black Fruit Wolfberry Biotechnology Co., LTD, Korla 841000, China

**Keywords:** Cantaloupe seed protein isolate, Ultrasound-assisted phosphorylation, Chitosan, Pickering emulsions, Nanoparticle

## Abstract

Cantaloupe seed protein isolate (CSPI) has attracted the attention of its low cost, easy digestion and balanced composition of essential amino acids. However, due to the low solubility of CSPI, its application in the food industry is limited. Therefore, the present study investigated the effect of ultrasound-assisted phosphorylation on the solubility of CSPI and the structural properties were characterized. The solubility of cantaloupe seed protein increased from 9.17 % to 63.27 % by ultrasound assisted phosphorylation, and resulting in an increase in the absolute value of CSPI potential, a decrease in particle size, and a stable structure, which could be used for the construction of the food emulsification system. The modified CSPI was combined with chitosan (CS) to prepare stabilized Pickering emulsion for subsequent stability study. The results showed that stable Pickering emulsions could be prepared with CSPI at pH 7, CS 0.5 % and oil phase fraction 55 %. Ultrasound-assisted phosphorylation enhanced electrostatic interaction between CS’s –NH_3_ groups and CSPI’s –COO−groups which improved the storability of stabilized Pickering emulsion. This will help to broaden the application range of CSPI and provide a theoretical basis for CPI stable Pickering emulsion.

## 1 Introduction

Plant protein is a rich source of dietary fibre and contains minimal amounts of cholesterol and saturated fat compared to animal protein. As a renewable resource, plants have a relatively short growth cycle, which allows them to be planted and harvested in a relatively short period of time. This aligns with the concept of green development, that minimizes environmental impact. As people become more focused on health and environmental protection, the status and significance of plant protein are also undergoing a process of constant enhancement [1]. During the processing of melon fruits, a considerable quantity of by-products is generated, among which the seeds of the cantaloupe are frequently discarded or as animal feed. Cantaloupe seeds are a rich source of protein and contain a diverse range of essential and non-essential amino acids. They are garnering increasing interest due to their significant nutritional and medicinal attributes [2]. The inherent characteristics of plant protein may not align with the requirements of specific food processing or applications. Modification can regulate the structure and function of protein, extend the storage life of protein and increase its stability, thereby rendering it suitable for a broader range of applications [3].

Ultrasonic modification refers to the use of 20–100 kHz ultrasonic wave frequency to modify massive proteins in a short time [4]. This method offers the dual advantages of high efficiency and low cost, which have attracted the attention of numerous researchers and have led to its widespread adoption in the field of food industry. Ultrasound treatment improved protein properties, for example, Xiong et al. [5] showed that ultrasonic treatment improved the foamability and foam stability of pea protein and the experimental results of this paper also proved that ultrasound expanded the protein structure, thereby improving the solubility of the protein, surface hydrophobicity and changing the surface charge, laying the foundation for the formation of a stable emulsion system. Sodium tripolyphosphate is a safe food additive permitted by the FDA. Protein phosphorylation is the selective reaction of phosphate groups with groups on the protein side chain [6], so that multiple negatively charged phosphate groups are introduced into the protein molecular structure, making it more electronegative. Phosphorylation can change the protein structure, thereby improving its solubility and emulsification [7]. Yan et al. [8] showed that phosphorylation changed the surface hydrophobicity and surface charge of walnut protein isolate, and increased the solubility. The emulsion activity index was increased by 2.5 to 4 times, the emulsion stability index was increased by up to 6 times, and the emulsion showed a relatively small size and large surface charge. Hu et al. [9] showed that the protein modified by ultrasound and phosphorylation showed the highest hydrophobicity and solubility, and the emulsifying activity and stability were significantly improved. As far as we know, the application of phosphorylation in protein modification has been applied in soybeans, peanuts, whey protein isolates, etc. However, there is currently no knowledge to evaluate the effect of phosphorylation on the structure of cantaloupe seed protein isolates (CSPI).

The traditional method of stabilizing emulsions is to add surfactants to emulsions, but these surfactants may cause some harmful reactions such as sensitization and carcinogenesis. Pickering emulsion is a new emulsion system, which is characterized by using solid particles instead of traditional organic surfactants to stabilize emulsions. It has stronger stability and biocompatibility, considering toxicity and food safety issues, people have turned their attention to food-grade solid particles such as polysaccharides and proteins as emulsifying stabilizers [10].

The effect of ultrasound-assisted phosphorylation on the structural characteristics of cantaloupe seed protein isolate (CSPI) has not been confirmed in the scientific literature. In this study, CSPI was selected as the research object, and its structural characteristics were characterized by ultrasonic modification, phosphorylation and ultrasound-assisted phosphorylation. In this paper, Pickering emulsion was formed between negatively charged cantaloupe seed protein and positively charged chitosan under phosphorylation conditions to enhance the stability of the emulsion, thus providing a theoretical basis for the application of cantaloupe seed protein in food, and evaluating its potential applications as nutritional and functional components.

## 2 Materials and methods

### 2.1 Materials

Cantaloupe seeds were provided by Xinjiang Black Fruit Wolfberry Biotechnology Co., LTD. The 1-anilino-8-naphthalenesulfonic acid (ANS) and spectroscopic grade potassium bromide (KBr) were obtained from Aladdin (Shanghai, China). Hexyl hydride, sodium tripolyphosphate, and other reagents used in this study were of analytical grade.

### 2.2 Preparation of cantaloupe seed protein isolate (CSPI)

The cantaloupe seeds are ground into a fine powder and placed in a cylindrical filter paper, and the filter paper roller is placed in a Soxhlet extractor. The samples were degreased with petroleum ether in a water bath at 70 °C, reflow for 8 h, and dried naturally for 12 h. Cantaloupe seed proteins were extracted by alkaline soluble acid precipitation [11]. The defatted powder was thoroughly mixed with water in the ratio of 1:10 (w/v), and the pH was adjusted to 9 with 3 M NaOH. Stirred for 1 h at room temperature and centrifuged (6000 r/min, 15 min). The supernatant was collected by centrifugation (6000 r/min, 15 min) after stirring for 1 h at room temperature and the pH was adjusted to 4.6 with the addition of 3 M HCl. The precipitate was collected and washed to neutrality with deionized water, and then freeze-dried to obtain the cantaloupe seed proteins isolated (CSPI).

### 2.3 CSPI and modification treatment

The CSPI powder was dissolved in NaOH solution (0.4 mg/L) to obtain 3 % (w/v) of CSPI, followed by the addition of 1 % (w/v) sodium trimetaphosphate, stirring for 20 min, and ultrasonication at 400 W, 20 kHz for 20 min followed by continuous phosphorylation in a water bath at 25 °C, after which the sample was immediately placed in an ice bath for 30 min to terminate the phosphorylation process. The samples were centrifuged (6000 r/min, 15 min), dialysed for 72 h and freeze-dried at 4 °C to obtain ultrasound-assisted phosphorylated cantaloupe seed protein (UP-CSPI). Phosphorylated samples without sonication and phosphorylated samples with sonication were referred to as so-called P-CSPI and U-CSPI, respectively.

### 2.4 Characterization of CSPI and modified group

#### 2.4.1 Solubility

The solubility was determined using the method of Zhang et al. [12]. A certain amount of sample freeze-dried powder was dissolved in sodium hydroxide aqueous solution (pH 9), fully hydrated, centrifuged (6000 r/min, 15 min), and an appropriate amount of supernatant was taken, and the protein concentration in the supernatant was determined by Coomassie brilliant blue G-250 by the spectrophotometer (UV-2450, Shimadzu, Japan). The solubility was obtained according to the measured protein content and the mass of the used protein powder. The solubility formula was as follows:

(1) $$C=\frac{C_{1}}{C_{2}}\times 100\%$$

where C_1_ was the protein content in the supernatant, mg/mL, and C_2_ was the theoretical concentration, mg/mL.

#### 2.4.2 Intrinsic fluorescence spectra analysis

The fluorescence spectra of the sample solution (0.1 mg/mL) were scanned by a fluorescence photometer (Lumina, Thermo Fisher Scientific, USA). The sample was scanned in the range of 300–400 nm, the excitation wavelength was 280 nm, the scanning speed was 1200 nm/min, the slit width was 5 nm, the scanning interval was 20 ms, and the reaction time was 0.1 s [13].

#### 2.4.3 Surface hydrophobicity (H_0_)

The H_0_ of proteins was characterized by the ANS fluorescence probe method, referring to Wen et al. [14]. The sample solution (4 mL, 0.05, 0.1, 0.15, 0.2, 0.25 mg/mL) was mixed with ANS (30 μL, 8 mM), and the reaction was dark for 15 min. The fluorescence intensity of the solution at the excitation wavelength of 390 nm and emission wavelength of 470 nm was measured by fluorescence spectrophotometer. The H_0_ was calculated by the slope of the fluorescence intensity and protein concentration curve.

#### 2.4.4 Zeta potential and particle size measurement

Zeta potential and particle size measurements of samples (0.1 mg/mL) were determined using a Zetasizer Nano (ZEN3700, Malvern, Britain). The Zetasizer Nano was set to Zeta potential mode and particle size mode. Zeta potential analysis was performed 20 times per group, and particle size analysis was performed 11 times per group [15].

#### 2.4.5 Free sulfhydryl (R-SH) and disulfide bond (R-S-S-R’) groups content

The levels of total and free sulfhydryl groups were determined by the Ellman method [16]. Determination of free sulfhydryl groups: 1 mL of sample solution was added to 5 mL of Tris-Gly buffer solution, followed by 0.1 mL of Ellman’s reagent. The mixture was then incubated at 25 °C for 15 min. The absorbance of the sample was measured at 412 nm. The solution without Ellman’s reagent was used as a blank control. Determination of total sulfhydryl groups: 1 mL of sample solution was added to 5 mL of Tris-gly-8 M Urea-0.5 % SDS buffer solution, followed by 0.1 mL of Ellman’s reagent. The disulfide bond content was calculated by the content of total sulfhydryl and free sulfhydryl.

(2) $$R-SH\;content\left(\mu mol/g\right)=\frac{73.53\times A_{412}}{C}$$

(3) $$Disulfide\;bond\;content\left(\mu mol/g\right)=\frac{Total\;sulfhydryl\;content-Free\;sulfhydryl\;content}{2}$$

where A_412_ was the absorbance at 412 nm, 73.53 was the conversion factor, and C was the sample concentration.

#### 2.4.6 Fourier transform infrared spectroscopy (FTIR) and secondary structure analysis

Referring to the method of Yan et al. [17], the freeze-dried powder and KBr were mixed at a ratio of 1:100, then ground evenly and pressed, scanned 32 times by Fourier Transform Infrared Spectrometer (Frontier, Perkinelmer, USA) in the range of 400–4000 cm^−1^.

#### 2.4.7 Contact angle measurement

Measured the contact Angle of the sample using a contact Angle meter (JC2000D1, Shanghai Zhongchen Digital Technology Equipment Co., LTD.). The freeze-dried sample powder was pressed into a thin slice with a thickness of 3 mm by a tablet press, and deionized water droplets were placed on the surface of the thin slice with a 5 μL microsyringe. The contact between the droplets and the sample was immediately photographed with a high-speed camera, and the Angle between the droplets and the sample was determined by the Angle method [10].

### 2.5 Preparation and characterization of UP-CSPI-CS composite nanoparticles

#### 2.5.1 Preparation of UP-CSPI-CS composite nanoparticles

The method of Zhao et al. [18] was referred and slightly modified. Chitosan (CS) was dissolved in 1 % (v/v) acetic acid solution and stirred at room temperature for 8 h to obtain CS reserve solution (1.2 wt%). UP-CSPI was mixed with CS solution of equal volume and different concentrations in equal proportion, ultrasonic 0.5 h, stirred 3 h, and ultrasound-assisted phosphorylation of cantaloupe seed protein-chitosan composite nanoparticles (UP-CSPI-CS) was obtained. The pH of the mixture was adjusted by 3 M HCl and 3 M NaOH to 3.0, 4.0, 5.0, 7.0, and 9.0, respectively. After ultrasound for 0.5 h (400 W, 20 kHz), stirred the mixture for 3 h. The final concentration of the final UP-CSPI was 1.5 %, and the final concentration of CS was 0.3, 0.4, 0.5, and 0.6 wt%, respectively.

#### 2.5.2 Turbidity of UP-CSPI-CS composite nanoparticles

With reference to the method and made slightly modified [19], and the samples were measured with an ultraviolet spectrophotometer with a wavelength of 600 nm.

#### 2.5.3 Zeta potential of UP-CSPI-CS composite nanoparticles

Referring to the method of Zhang et al. [20], after diluting the sample solution 150 times, the Zetasizer Nano was used for determination, appropriate samples were added to the measuring pool, adjusted to Zeta potential mode, and balanced for 120 s (25 °C) before measurement. Zeta potential analysis was performed 20 times for each group.

### 2.6 Preparation and characterization of UP-CSPI-CS composite nanoparticles stabilized Pickering emulsion

#### 2.6.1 Preparation of Pickering emulsion

The prepared UP-CSPI-CS solution (pH 7.0) was 15 mL, added with 5 mL soybean oil, homogenized in a homogenizer at 12,600 r/min for 2 min, 400 W ultrasound for 20 min, and the resulting Pickering emulsion was transferred to a glass bottle with sodium azide (0.02 %, w/v) and stored at 4 °C for further study.

#### 2.6.2 Characterization of Pickering emulsion

##### 2.6.2.1 Particle size determination of Pickering emulsion

The prepared UP-CSPI-CS emulsion was measured with the Zetasizer Nano. Appropriate samples were added to the measuring tank, adjusted to the particle size mode, and balanced for 120 s (25 °C) before measurement. The particle size analysis of each group was run 11 times.

##### 2.6.2.2 Measurement of Pickering emulsion rheological properties

Referring to the method of Zhang et al. [20], the Anton Parr rheometer was used to characterize the apparent viscosity of the prepared sample in the shear rate range of 0.1 to 100 s^−1^. A PP50 mm flat plate was used for measurement. After the flat plate was corrected for zero gap, the measurement program was set. Took about 3 mL of solution to the fixture gap, kept all tested samples at 25 °C, and balanced for 5 min before measurement. The viscosity of different samples was determined and each treatment was repeated three times.

##### 2.6.2.3 Determination of Pickering emulsion microstructure

The emulsion with oil phase ratios of 35 %, 45 %, 55 %, 65 %, and 75 % was diluted 10 times and dropped on a slide. The images were recorded with a microscope under a 40 × objective lens. 10 images were taken for each sample, and the images with universal characteristics were selected [21].

##### 2.6.2.4 Determination of creaming index

The delamination stability of the emulsion was indicated by the creaming index (CI) [22]. The CI value was calculated by the following formula:

(4) $$CI\left(\%\right)=\frac{H_{s}}{H_{t}}\times 100$$

H_s_ was the bottom cleaning height of the sample bottle; H_t_ was the total height of the emulsion.

### 2.7 Stability of Pickering emulsion

#### 2.7.1 Oil phase stability

The prepared UP-CSPI-CS solution was mixed with soybean oil so that the oil phase ratio was 35 %, 45 %, 55 %, 65 %, and 75 %, homogenized in a homogenizer at 12,600 r/min for 2 min, and the resulting Pickering emulsion was transferred to a glass bottle and stored at 4 °C for further study.

#### 2.7.2 Storage stability

The prepared UP-CSPI-CS solution was mixed with soybean oil with an oil phase ratio of 55 % and homogenized in a homogenizer at 12,600 r/min for 2 min. The resulting Pickering emulsion was transferred to glass bottles and stored at 4 °C for further study.

#### 2.7.3 Thermal stability

The prepared UP-CSPI-CS solution was mixed with soybean oil, the oil phase ratio was 55 % and homogenized in a 12,600 r/min homogenizer for 2 min. The Pickering emulsion was transferred into glass bottles and treated at 4, 25, 40, 65, 90 °C for 30 min, then stored at room temperature for further study.

#### 2.7.4 Ionic strength stability

The prepared UP-CSPI-CS solution was mixed with soybean oil, the oil phase ratio was 55 %, the NaCl concentration was 0.1, 0.2, 0.3, 0.4 mol, respectively. Then 12,600 r/min homogenized for 2 min. The resulting Pickering emulsion was transferred to glass bottles and stored at room temperature for further study.

### 2.8 Data processing

All experiments were repeated three times, and the mean value was taken. The experimental data were expressed in the form of mean ± standard deviation. SPSS 21.0 software was used for Duncan’s significance difference analysis, and *p* < 0.05 was used as the significance test standard.

## 3 Results and discussion

### 3.1 Characteristics of CSPI and modified group

#### 3.1.1 Solubility analysis

**Fig. 1:**
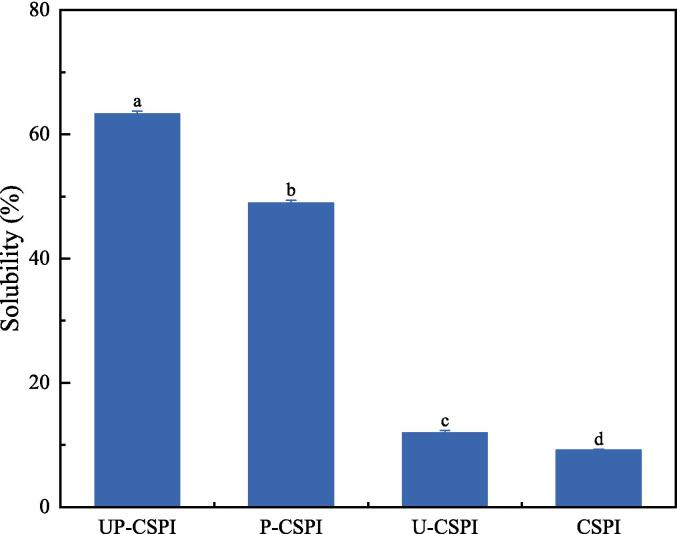
Solubility of CSPI under different modification treatments (CSPI: cantaloupe seed protein isolate; U-CSPI: CSPI only ultrasound; P-CSPI: CSPI only phosphorylation; UP-CSPI: CSPI ultrasound-assisted phosphorylation).

Solubility is a key factor affecting the functional properties of proteins and can regulate many other related properties, such as emulsification, foaming, and gelling [9]. As shown in Fig. 1, CSPI had a low solubility of 9.17 %, both ultrasound and phosphorylation can significantly improve the solubility of CSPI, and the solubility of UP-CSPI was the highest (63.27 %), which was significantly higher than other levels (*p* < 0.05), which was consistent with the research results of Hu et al. [9], they found that the solubility of goose liver protein treated with ultrasonic-assisted phosphorylation increased the most (33.53 %). This was because ultrasound can accelerate the collision of protein molecules, causing the change of protein conformation, and ultrasound can unfold proteins, making them more soluble [23]. In addition, phosphate groups attached to protein molecules can form hydrogen bonds with water molecules, increasing the electrical repulsion between protein molecules, and thus promoting the binding between protein and water molecules [24]. In addition, Ren et al. [25] showed that the introduction of phosphate groups into proteins could increase the electronegativity of proteins, especially on their surfaces, and generate higher electrical repulsion, which was conducive to the improvement of CSPI solubility.

#### 3.1.2 Intrinsic fluorescence spectrum scanning analysis

**Fig. 2:**
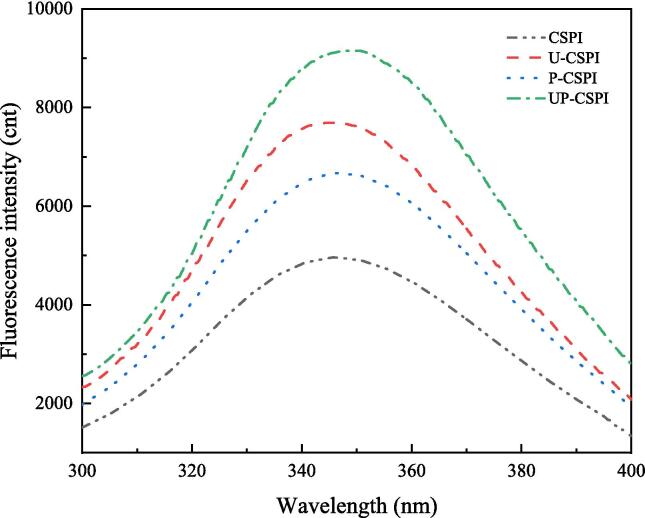
Fluorescence intensity of CSPI under different modified treatments.

The peak position and intensity of the intrinsic fluorescence spectrum are correlated with the environment surrounding the tryptophan residues present in the protein [26]. In general, the higher the fluorescence intensity, the higher the degree of protein unfolding and tryptophan residue exposure [27]. CSPI had the lowest fluorescence intensity, followed by P-CSPI (Fig. 2). Phosphoric acid groups have a strong steric hindrance effect, which may lead to a decrease in fluorescence intensity [28]. The fluorescence intensity of U-CSPI and UP-CSPI were higher, because that ultrasonic wave promoted the CSPI molecule to unfold, exposing hidden tryptophan residues and hydrophobic groups to the microenvironment, thus increasing the fluorescence intensity [26].

#### 3.1.3 Surface hydrophobicity (H_0_) analysis

**Fig. 3:**
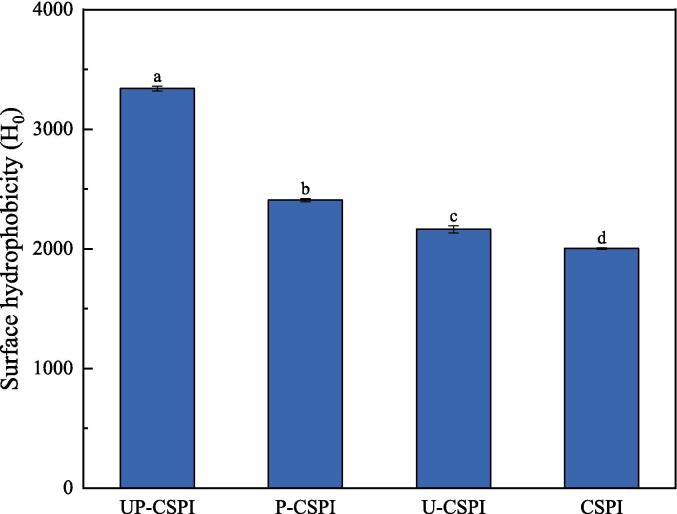
Surface hydrophobicity of CSPI under different modification treatments.

H_0_ is an important factor in maintaining protein conformation [29] and also affects many functional properties [8]. Fig. 3 showed UP-CSPI exhibited the highest H_0_, while CSPI had the lowest H_0_. This was because ultrasound can make protein structure more porous, accelerate molecular collision, and expose the hydrophobic groups buried in CSPI molecules, thus increasing the H_0_. After phosphorylation, the surface charge of CSPI will be changed, the electrostatic repulsion between droplets will increase, and the droplets will be more easily dispersed, which may change the secondary and tertiary structure of proteins, and further expose the hydrophobic residues on the molecular surface of CSPI [30], and phosphate and sodium ions were easy to bind to proteins. This results in conformational changed of CSPI and increased H_0_ [31]. In addition, ultrasound and phosphorylation had a synergistic effect in improving the H_0_ of CSPI, exposing more internal hydrophobic groups and resulting in higher H_0_, which was consistent with the research results of Hu et al. [9].

#### 3.1.4 Zeta potential analysis

**Fig. 4:**
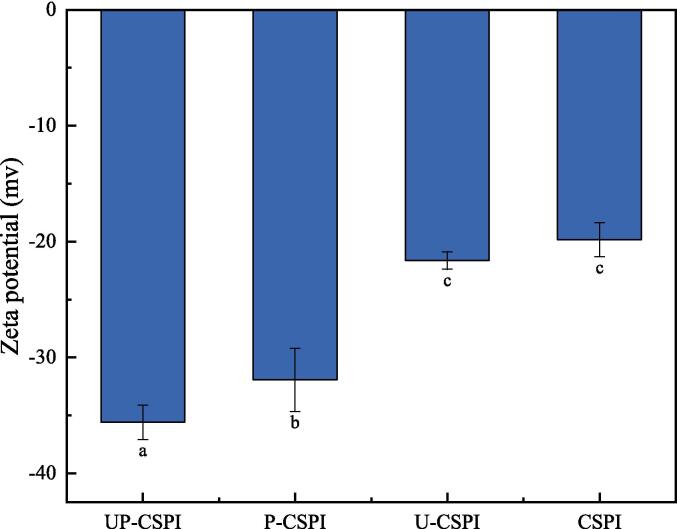
Zeta potential of different modified CSPI.

The Zeta potential can be used to indicate the stability of a solution system, affecting its dispersion and aggregation [32]. Generally, the higher the absolute value of zeta potential (≥30 mV) of the solution system, the higher the electrostatic repulsion force, the stability of the solution is high, and there is less flocculation and aggregation [33]. Fig. 4 showed the potential of UP-CSPI was −35.60 mv, which was significantly higher than other levels (*p* < 0.05). Ultrasound could expand CSPI molecules and expose more phosphorylation sites, which was the key to enhancing the interaction between protein molecules and phosphate groups and promoting phosphorylation. This was consistent with the results of surface hydrophobicity, which indicated that ultrasound-assisted phosphorylation had a positive effect on CSPI.

#### 3.1.5 Particle size distribution analysis

**Fig. 5:**
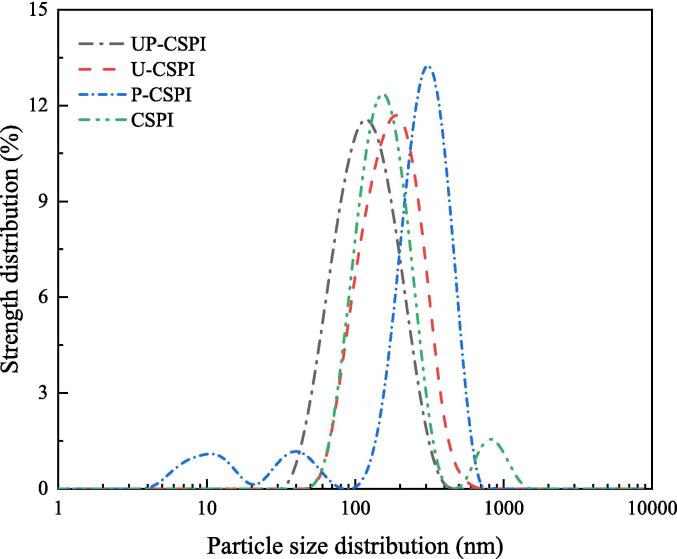
Particle size distribution of CSPI under different modification treatments.

Particle size is an important indicator of protein, which has a certain impact on the functional properties of protein [34]. In Fig. 5, CSPI, U-CSPI, P-CSPI, and UP-CSPI exhibited different particle size distributions. CSPI presented a larger particle size and multi-peak form. After ultrasonic treatment, the cavitation effect of ultrasound broke the non-covalent bond between particle molecules, dissociated CSPI aggregates, and reduced the particle size of the complex [33]. Phosphorylation changed the charge distribution on the surface of CSPI, increased the negative charge, reduced the surface tension of the liquid, made adjacent droplets have a strong electrostatic repulsion force, and prevented the formation of droplet aggregation, which effectively reduced the particle size of CSPI. UP-CSPI showed the lowest particle size distribution, and the results showed that ultrasound-assisted phosphorylation had a positive effect on CSPI.

#### 3.1.6 Sulfhydryl group and disulfide bond content analysis

**Fig. 6:**
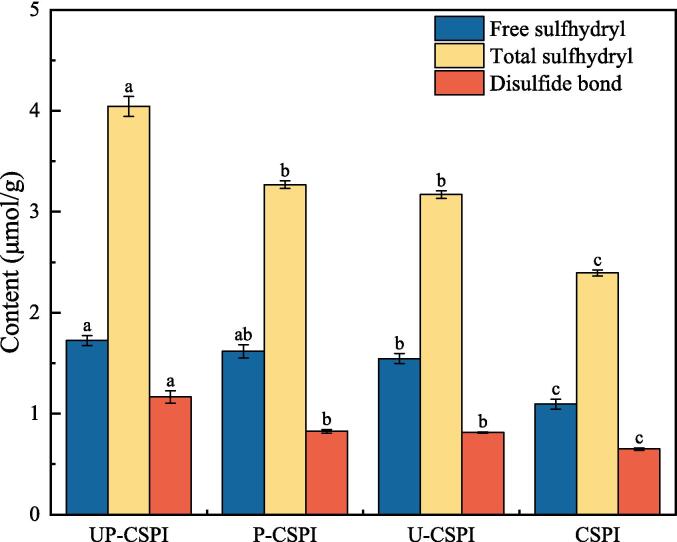
Contents of sulfhydryl group and disulfide bond in CSPI treated with different modifications.

The increase in sulfhydryl group content may be due to the unfolding of the protein structure or the break of the disulfide bond. The conversion between sulfhydryl and disulfide bonds will lead to changes in protein structure, and its content can affect the stability of spatial structure and thus affect the functional properties of proteins [35]. Fig. 6 showed that the content of free sulfhydryl in CSPI was 1.09 μmol/g, and the content of free sulfhydryl in modified samples was significantly increased (*p* < 0.05), which may be due to the disintegration of CSPI structure caused by modified treatment [36], which exposed the internal sulfhydryl and converted it into free sulfhydryl. Disulfide bonds of CSPI were cleaved to form new free sulfhydryl groups [37].

#### 3.1.7 FTIR analysis

**Fig. 7:**
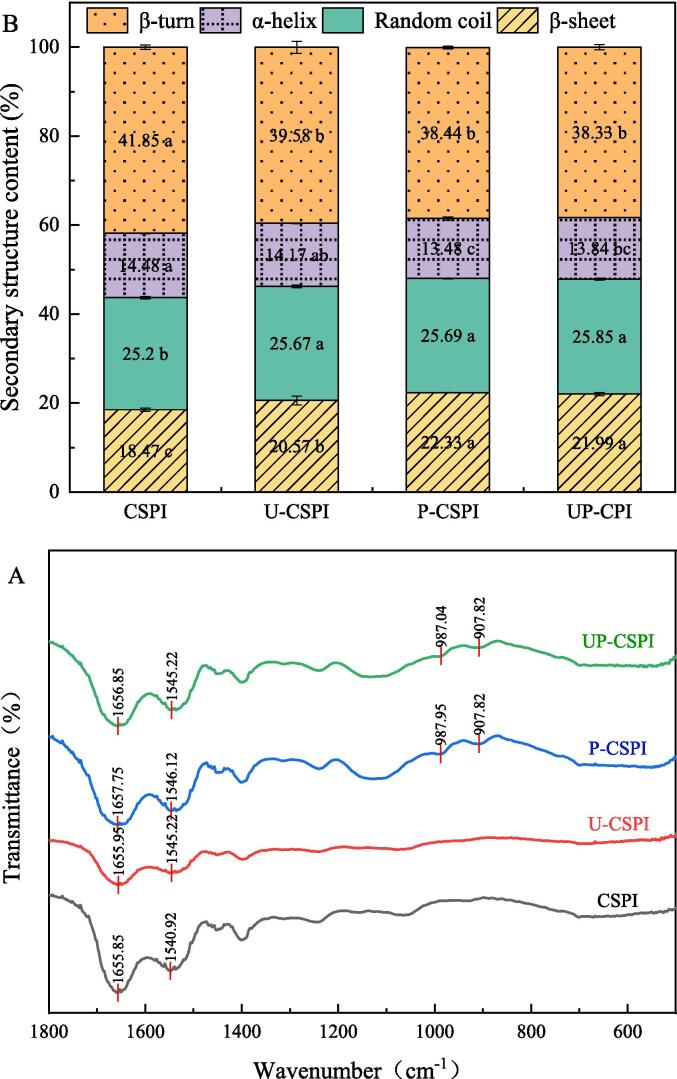
(A) Infrared spectrum scanning and (B) secondary structure of CSPI under different modification treatments.

Fig. 7(A) showed FTIR of CSPI under different modification treatments. There were obvious differences between U-CSPI, P-CSPI, UP-CSPI, and CSPI. The peak positions of the two bands of CSPI were 1655.85 cm^−1^ and 1540.92 cm^−1^, respectively. New absorption bands with wave numbers of 987 cm^−1^ and 907 cm^−1^ were also shown in P-CSPI and UP-CSPI, which were identification bands for P = O and P-O. The presence of absorption bands for P = O and P-O indicated that the phosphate group had been successfully introduced into the protein.

FTIR can calculate the content of secondary structures. The main absorption bands of proteins were the amide I band (1700–1600 cm^−1^, mainly due to C-O stretching vibrations of peptide bonds) and the amide II band (1600–1500 cm^−1^, including N-H bending vibrations and C-N stretching vibrations) [38]. In Fig. 7 (B), the content of U-CSPI, P-CSPI, UP-CSPI β-sheet structure, and random coil structure increased (*p* < 0.05), while the contents of α-helix and β-turn structure were decreased (*p* < 0.05), indicating that the secondary structure of the protein was changed by modification, the decrease of α-helix structure and the increase of irregular curly structure mean that the secondary structure of the protein changed from relatively ordered to relatively disordered direction, which will reduce the overall rigidity of the protein and increase the flexibility.

#### 3.1.8 Contact Angle analysis

**Fig. 8:**
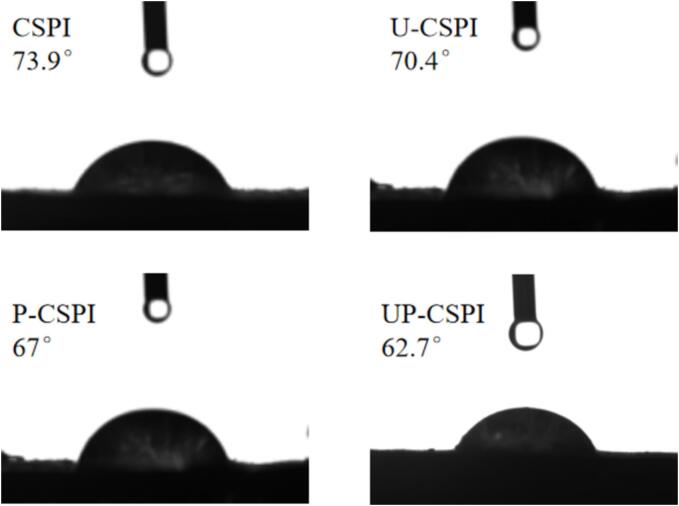
Contact Angle of different modified CSPI.

In Fig. 8, the contact angle of CSPI was 73.9°. After modification, the contact angle all decreased, among which the contact angle of UP-CSPI was the smallest, and it had high hydrophilicity. The reason may be that a large number of hydrophilic hydroxyl groups were introduced in the phosphorylation process, and ultrasound can accelerate the collision of protein molecules and cause the change in protein conformation [23]. With ultrasound-assisted phosphorylation, phosphate groups can form a large number of hydrogen bonds with water molecules, which increased the electrical repulsion between CSPI molecules, thus promoting the binding between CSPI and water molecules and showing high wettability [24].

### 3.2 Characteristic of UP-CSPI-CS composite nanoparticles

#### 3.2.1 Turbidity analysis of UP-CSPI-CS composite nanoparticles

**Fig. 9:**
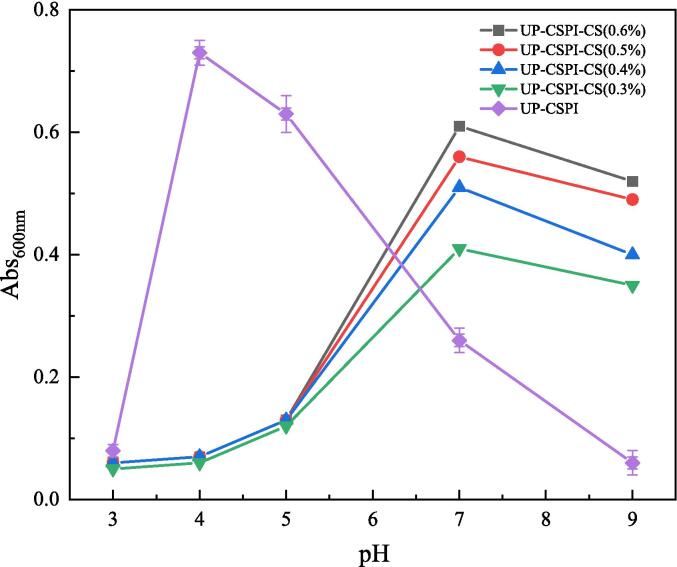
Turbidity of UP-CSPI-CS complex solution at different pH.

In Fig. 9, the turbidity changed of the complex solution formed by UP-CSPI and CS of different concentrations in equal volume ratios at different pH values were shown. The mass ratio of polysaccharide to protein has a high influence on the formation of polysaccharide-protein complex [39]. With the increase in pH value, the absorbance of the UP-CSPI-CS composite solution changed. According to the change of turbidity, it can be divided into four regions: A (cosoluble zone), B (soluble zone), C (miscible zone), and D (coinsoluble zone). In zone A, pH < 4, the turbidity of the composite solution hardly changed, indicating that there was no interaction between UP-CSPI and CS [40]; in zone B, pH 4–6, the turbidity of the solution increased with the increase of pH, indicating that the negatively charged carboxyl group on UP-CSPI binds with the positively charged amino group on CS to form a soluble complex in this region. In the C region, pH 6–8, the turbidity increased rapidly, and at pH_opt_ (7), the turbidity was the largest, indicating the formation of an insoluble complex. In the D region, when pH > 8, this was because the pH value of the composite system was close to the pKa value of chitosan, and the degree of protonation was reduced, so the system was cloudy.

#### 3.2.2 Zeta potential analysis UP-CSPI-CS composite nanoparticles

**Fig. 10:**
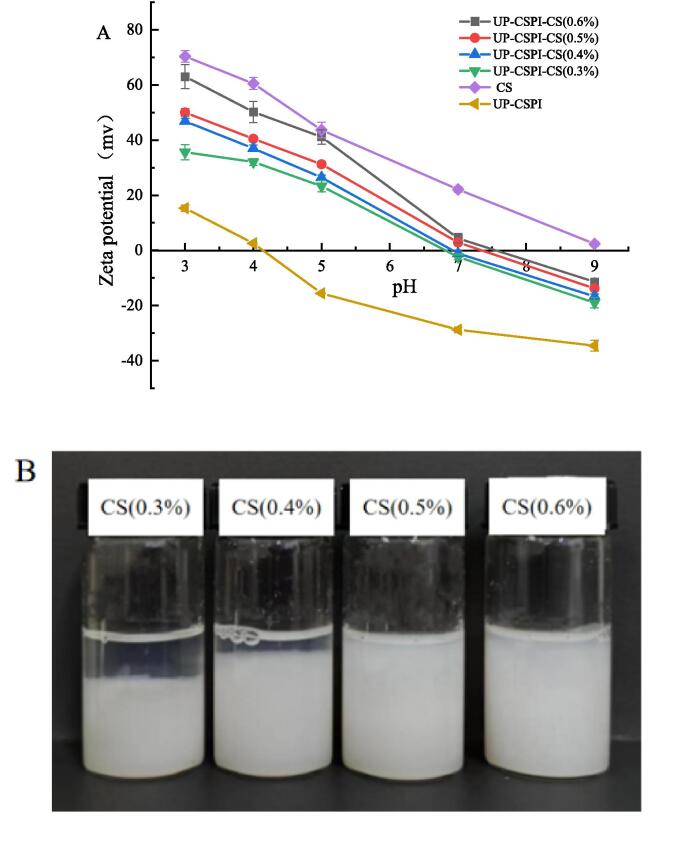
(A) Zeta potential and (B) topography of UP-CSPI-CS complex solution with different pH.

As shown in Fig. 10(A), to further analyze the interaction between UP-CSPI and CS, the Zeta potential changes of the complex solution formed by UP-CSPI and CS with different concentrations in equal volume ratios at different pH values were tested. The Zeta potential of all samples decreased with the increase of pH value, and the Zeta potential value of the UP-CSPI-CS composite solution was about zero at pH 7.0, and the CS solution was positively charged within the range of pH 3–9, which was similar to the research results of Dong et al. [39]. The complex coacervation of UP-CSPI and CS was driven by electrostatic interaction. When the pH value is low, the soluble complex is formed because the UP-CSPI composite solution is positively charged, and the electrostatic repulsion prevents further aggregation among particles. With the increase of pH value, the protein itself carries a negative charge, while CS always carries a positive charge, and the complex carries a reduced charge. Enhanced electrostatic attraction led to aggregation and precipitation of the UP-CSPI-CS complex [39]. When pH = pH_opt_ (7), the composite condensation effect was the strongest. In addition, in Fig. 10 (B), the stability of the UP-CSPI-CS composite solution was observed after it was placed at room temperature for 24 h at pH 7.0. No stratification occurred at 0.5 % and 0.6 % concentrations of the composite solution, indicating that the UP-CSPI-CS composite solution had high stability under this condition. Then the next experiments were carried out with 0.5 % CS solution.

### 3.3 Characteristic of UP-CSPI-CS Pickering emulsion

#### 3.3.1 Rheological analysis of UP-CSPI-CS Pickering emulsion

**Fig. 11:**
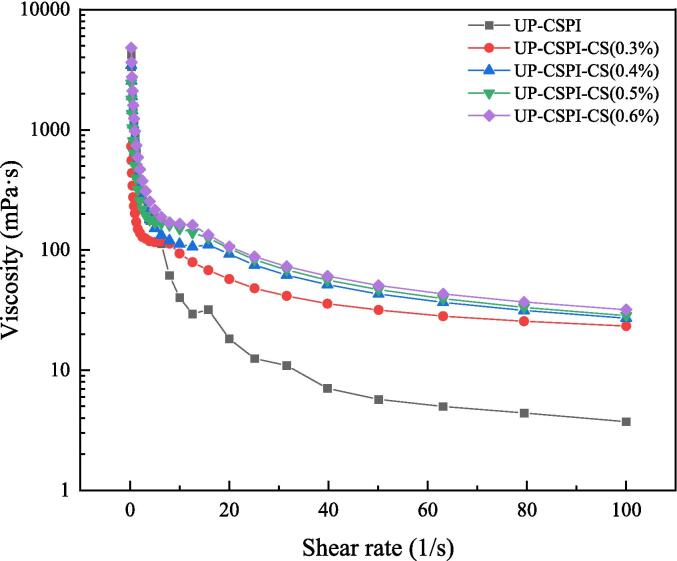
The viscosity of UP-CSPI-CS stable Pickering emulsion with shear rate.

Rheology is important for the study of the stability and microstructure of Pickering emulsions. In Fig. 11, the properties of Pickering emulsions with 25 % oil phase fraction under different concentrations of CS (0.3 %, 0.4 %, 0.5 %, 0.6 %) at room temperature were studied, and all of them were gel-like network structures with elastic behavior. As the amount of CS increased, its viscosity showed a trend of first increasing and then decreasing, indicating that the intramolecular structure formed by the electrostatic interaction between UP-CSPI and CS was denser than that formed between protein molecules, and the network structure formed by electrostatic interaction also hindered the shear to a certain extent [41]. These results also proved that the enhancement of rheological properties of the UP-CSPI-CS composite condensate was due to the electrostatic interaction between biomacromolecules [42].

#### 3.3.2 Particle size analysis of UP-CSPI-CS stable Pickering emulsion

**Fig. 12:**
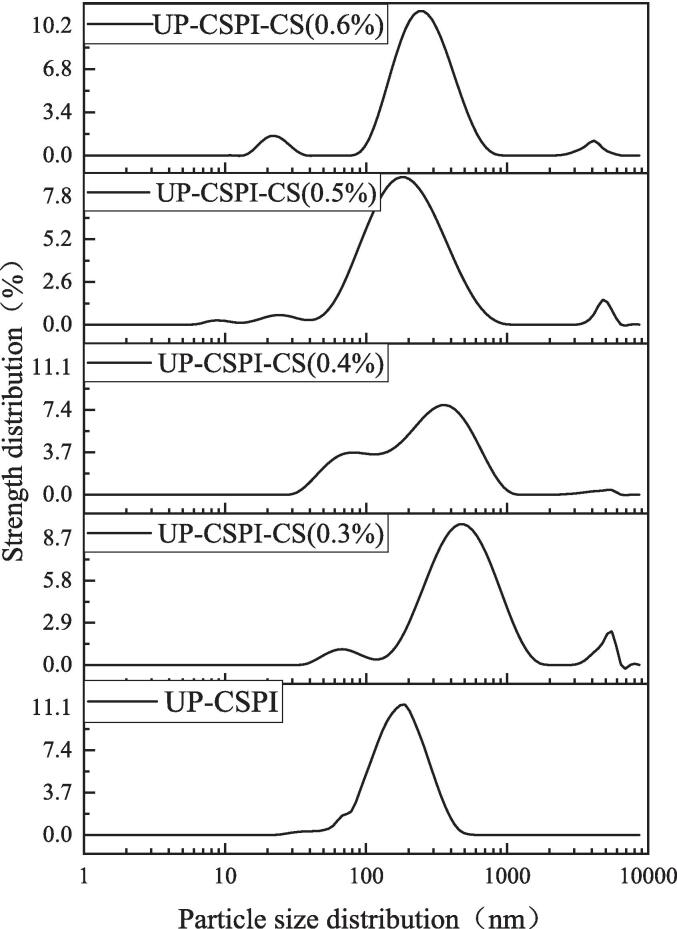
Particle size distribution of UP-CSPI-CS stable Pickering emulsion.

In Fig. 12, the particle size distribution of Pickering emulsions with 25 % oil phase fraction under different concentrations of CS (0.3 %, 0.4 %, 0.5 %, 0.6 %) at room temperature was studied. UP-CSPI as the control, it can be seen from Table 1. That with the increase of CS concentration, the droplet size of Pickering emulsion decreased significantly (*p* < 0.05), from 3726.43 nm to 1346.75 nm. This was because the increase in particle concentration at the oil–water interface reduced the free energy, thus stabilizing the entire system. This was consistent with the results of Zhao et al. [43]. Higher particle concentration and smaller particle size were conducive to improving the adsorption at the oil–water interface, forming an interface film, increasing the steric hindrance between droplets, thereby preventing droplet aggregation and improving the stability of the emulsion.

**Table 1:** Average particle size of UP-CSPI-CS stable Pickering emulsion.

Chitosan concentration	Partical size (nm)
UP-CSPI	684.43 ± 36.54^d^
UP-CSPI-CS(0.3 %)	3726.43 ± 132.56^a^
UP-CSPI-CS(0.4 %)	1967.43 ± 74.44^b^
UP-CSPI-CS(0.5 %)	1354.75 ± 61.76^c^
UP-CSPI-CS(0.6 %)	1346.75 ± 54.64^c^

Note: Different superscript letters in the table indicate significant differences (*p* < 0.05).

#### 3.3.3 Oil comparison for emulsion stability analysis

**Fig. 13:**
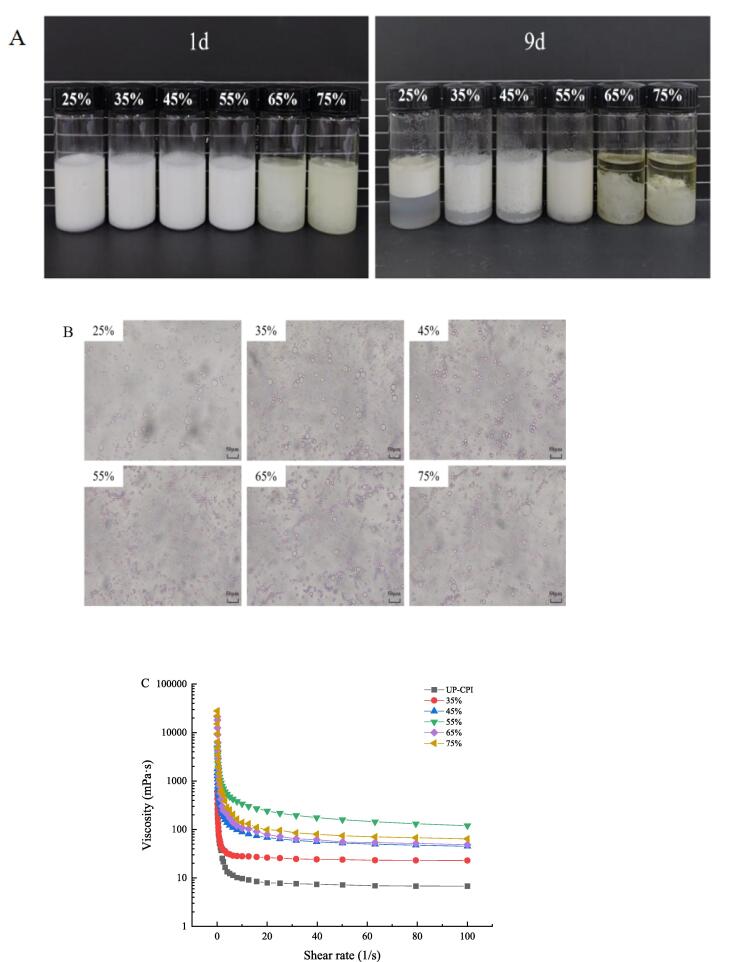
Morphology (A), microstructure (B), apparent viscosity versus shear rate curve (C) of UP-CSPI-CS stabilized Pickering emulsion with different oil phases.

In Fig. 13, the properties of Pickering emulsions with CS concentration of 0.5 % under different oil phase mass fractions φ (25 %, 35 %, 45 %, 55 %, 65 %, 75 %, v/v) were studied. Pictures of different oil phase UP-CSPI-CS complex emulsions taken over 9 days in Fig. 13 (A). When the oil phase fraction was 25 %, 35 %, and 45 %, the water phase was separated from the bottom, and when the oil phase fraction was 65 % and 75 %, the oil phase was separated from the upper end of the emulsion. This phenomenon can be explained by the fact that the UP-CSPI-CS complex particles in the continuous phase were not enough to disperse oil in the whole system. When the emulsifier is insufficient to cover the interface, flocculation occurs [44], resulting in the separation of the oil and water phases. When the oil phase content was 55 %, a uniform emulsion was obtained, and it also remained stable during storage at room temperature for 9 days. This indicated that the UP-CSPI-CS complex had high delamination resistance during storage at room temperature.

It was well known that droplets with smaller droplet sizes have higher stability [45]. At the same time, it can be seen from the microstructure (Fig. 13 (B)) of the emulsion that when the oil phase content was 55 %, the droplet size was small, the distribution was uniform, the distribution was dense and the shape was smooth. The emulsion existed as dispersed droplets in a continuous phase, which was more stable and had a relatively long shelf life[46]. From rheology (Fig. 13(C)), it can be seen that when the oil phase content was 55 %, there was the highest viscosity, the emulsion interaction was the strongest at this time, and the emulsion stability was the highest. Among them, the viscosity of the emulsion in the control group and 35 % was the lowest, and combining Fig. 13(A) and Fig. 13(B) can conclude that the emulsion was unstable under this condition and was most easily stratified. The optimal condition for preparing UP-CSPI-CS particle-stabilized Pickering emulsion was the 55 % oil phase fraction. This was similar to the results of Yu et al. [41].

#### 3.3.4 Stability analysis of emulsion for storage time

**Fig. 14:**
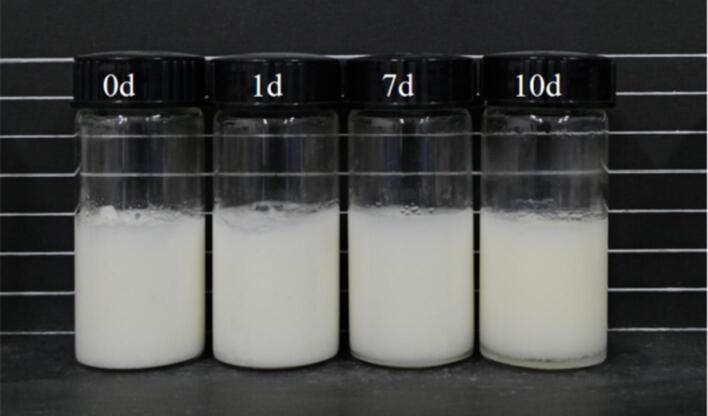
Morphology of UP-CSPI-CS of stable Pickering emulsion for different storage times.

The properties of Pickering emulsion at room temperature with 0.5 % CS and oil phase fraction of 55 % for different storage times (0 d, 1 d, 7 d, 10 d) were studied. The Fig. 14, showed a picture of different oil phase UP-CSPI-CS complex emulsions taken over 10 days, with the bottom water phase separated at room temperature on day 10 of storage. At the same time, the particle size of the emulsion with different storage days was studied. As can be seen from Table 2, the particle size of the emulsion increased significantly with the increase of storage days (*p* < 0.05), because with the extension of storage time, the interaction force between the UP-CSPI-CS complex weakened, and the droplet appeared flocculation and aggregation, thus increasing the particle size.

**Table 2:** The particle size of UP-CSPI-CS stable Pickering emulsion for different storage times.

Storage days	Partical size (Nm)
0 d	1102.23 ± 54.24^d^
1 d	1639.83 ± 136.13^c^
7 d	6421.32 ± 313.74^b^
10 d	7231.73 ± 417.33^a^

Note: Different superscript letters in the table indicate significant differences (*p* < 0.05).

#### 3.3.5 Temperature analysis of emulsion stability

**Fig. 15:**
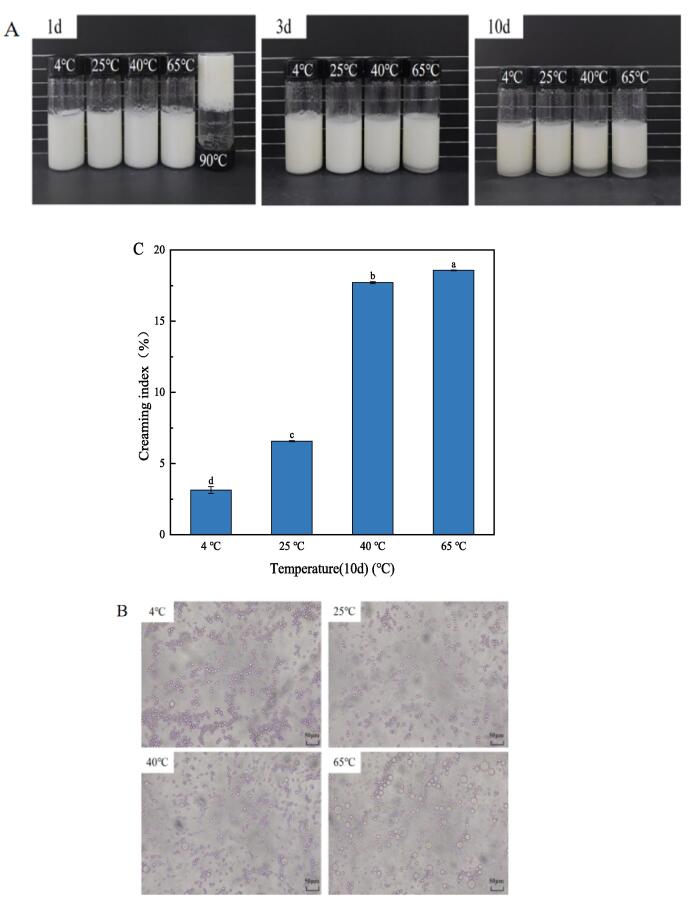
(A) Morphology, (B) microstructure, (C) creaming index of UP-CSPI-CS stabilizes Pickering emulsion at different temperatures.

In Fig. 15, the properties of Pickering emulsion at room temperature with 0.5 % CS and 55 % oil phase fraction at different heat treatment temperatures (4, 25, 40, 65, 90 °C) were studied. The picture of the UP-CSPI-CS complex emulsion taken at different temperatures over 10 days was shown in Fig. 15(A). Under other temperature treatments, the samples were emulsion, but after treatment at 90 °C, the sample was a non-flowing solid, which may be due to the formation of gel induced by heat. Samples treated at 40 °C and 65 °C began to stratify on day 3, with water phase precipitation. In Fig. 15(C), on the 10th day, the CI at 40 °C and 65 °C was 17.71 % and 18.57 %, respectively. The samples treated at 4 °C and 25 °C began to delaminate, and the CI was 3.14 % and 6.57 %, which was because after heat treatment, the interaction force between various components was disrupted and the original equilibrium state was lost. This affects the stability of the whole system. As can be seen from the microscopic structure diagram in Fig. 15(B), large droplets, flocculation, and aggregation occurred especially in the samples treated at 65 °C. Therefore, heat treatment was an important factor affecting the stability of the complex.

#### 3.3.6 Analysis of ionic strength on emulsion stability

**Fig. 16:**
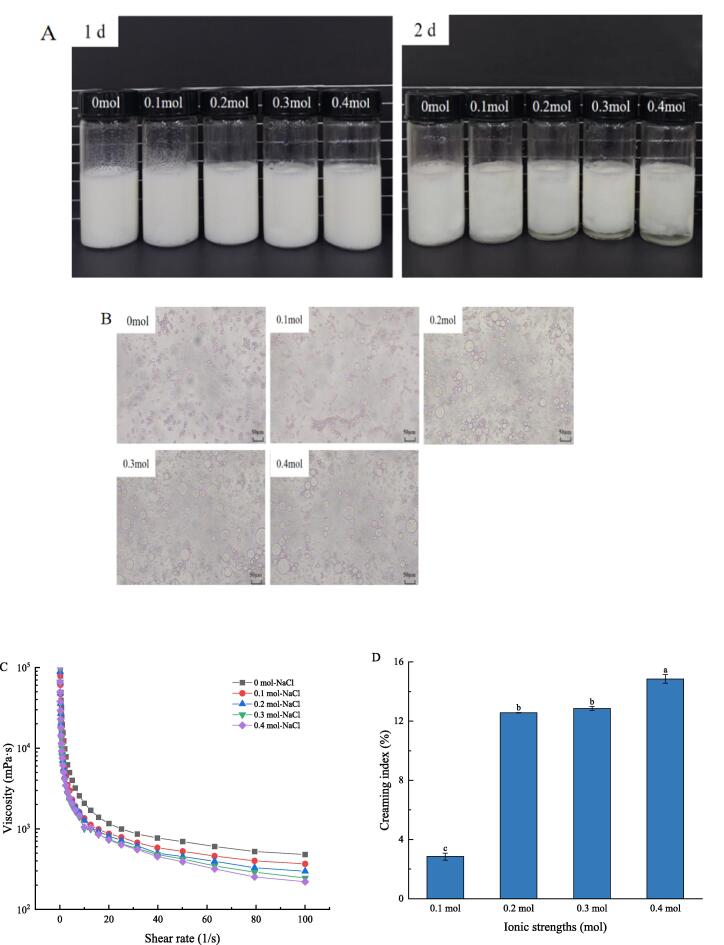
(A) Topography, (B) microstructure, (C) viscosity versus shear rate curve, (D) creaming index of UP-CSPI-CS stabilizes Pickering emulsions with different ionic strengths.

The ionic strength plays an important role in controlling the charge balance of the electrostatic complex system [47]. NaCl can not only reduce the surface potential of colloidal particles, but also cause flocculation and precipitation of particles. Therefore, it is valuable to study the stability of Pickering emulsion stabilized by UP-CSPI-CS nanoparticles under certain ionic strength. Fig. 16(A) showed the visual appearance of the UP-CSPI-CS complex under different concentrations of NaCl. Pickering emulsion with UP-CSPI-CS nanoparticle concentration of 0.5 wt% and oil phase of 55 % exhibited the highest level of stability and on this basis, different concentrations of NaCl were added. As the concentration of NaCl increased, the stratification of the emulsion became increasingly pronounced. As shown in Fig. 16(D), when NaCl concentration was 0.4 mol, the CI reached 14.85 %. At the same time, it can be seen from Fig. 16(B) that the emulsion droplets increased and aggregated, and this indicated that the addition of NaCl weakened the electrostatic interaction and adversely affected the stability of the complex [48].

Rheological property is one of the important indexes to characterize the stability and function of Pickering emulsion. It can be seen from Fig. 16(C) that all emulsions exhibited shear thinning characteristics, and the apparent viscosity decreased with the increase of shear rate, indicating that the adhered oil droplets disperse under the action of shear force, confirming the existence of bridge flocculation structure in the emulsions [41]. With the increase of NaCl concentration, the viscosity of the emulsion decreased, and it can be considered that electrostatic interaction was the main force in maintaining the structural cohesion between protein and polysaccharide [47].

## 4 Conclusions

This study demonstrated that ultrasound-assisted phosphorylation induced molecular structure changes of CSPI, resulting in a decrease in the particle size of CSPI molecules, an increase in fluorescence intensity and surface hydrophobicity, and ultimately an increase in the solubility of CSPI. In addition, ultrasound-assisted phosphorylated cantaloupe seed protein-chitosan stabilized Pickering milk had a smaller, more uniform droplet distribution, higher stability to environmental stresses (temperature, ionic concentration, and pH), and higher emulsion delamination stability. These results suggested that the combination of CSPI with chitosan after ultrasound-assisted phosphorylation can improve its emulsion stability, underscoring its potential as a stabilizer for milk-based product development.

## CRediT authorship contribution statement

**Ruihua Zhang:** Writing – review & editing, Data curation. **Shuting Li:** Writing – review & editing, Data curation. **Mingyan Ai:** Visualization, Conceptualization. **Shenghuizi Chen:** Investigation, Formal analysis. **Chunlan Zhang:** Methodology, Conceptualization. **Zhiqiang Zhou:** Software. **Lili Huang:** Software. **Xiang Li:** Resources. **Jiankang Lu:** Writing – review & editing, Methodology.

## Declaration of competing interest

The authors declare the following financial interests/personal relationships which may be considered as potential competing interests: Jiankang Lu reports financial support was provided by the National Natural Science Foundation of China (No.31860431). If there are other authors, they declare that they have no known competing financial interests or personal relationships that could have appeared to influence the work reported in this paper.
